# Prolonged course of brain edema and neurological recovery in a translational model of decompressive craniectomy after closed head injury in mice

**DOI:** 10.3389/fneur.2023.1308683

**Published:** 2023-11-20

**Authors:** Jacek Szczygielski, Vanessa Hubertus, Eduard Kruchten, Andreas Müller, Lisa Franziska Albrecht, Karsten Schwerdtfeger, Joachim Oertel

**Affiliations:** ^1^Department of Neurosurgery, Saarland University Medical Center and Saarland University Faculty of Medicine, Homburg, Germany; ^2^Instutute of Neuropathology, Saarland University Medical Center and Saarland University Faculty of Medicine, Homburg, Germany; ^3^Institute of Medical Sciences, University of Rzeszów, Rzeszow, Poland; ^4^Department of Neurosurgery, Charité University Medicine, Berlin, Germany; ^5^Berlin Institute of Health at Charité, Berlin, Germany; ^6^Institute of Interventional and Diagnostic Radiology, Karlsruhe, Germany; ^7^Department of Radiology, Saarland University Medical Center and Saarland University Faculty of Medicine, Homburg, Germany

**Keywords:** mouse model, brain edema, decompressive craniectomy, traumatic brain injury, magnetic resonance imaging, closed head injury

## Abstract

**Background:**

The use of decompressive craniectomy in traumatic brain injury (TBI) remains a matter of debate. According to the DECRA trial, craniectomy may have a negative impact on functional outcome, while the RescueICP trial revealed a positive effect of surgical decompression, which is evolving over time. This ambivalence of craniectomy has not been studied extensively in controlled laboratory experiments.

**Objective:**

The goal of the current study was to investigate the prolonged effects of decompressive craniectomy (both positive and negative) in an animal model.

**Methods:**

Male mice were assigned to the following groups: sham, decompressive craniectomy, TBI and TBI followed by craniectomy. The analysis of functional outcome was performed at time points 3d, 7d, 14d and 28d post trauma according to the Neurological Severity Score and Beam Balance Score. At the same time points, magnetic resonance imaging was performed, and brain edema was analyzed.

**Results:**

Animals subjected to both trauma and craniectomy presented the exacerbation of the neurological impairment that was apparent mostly in the early course (up to 7d) after injury. Decompressive craniectomy also caused a significant increase in brain edema volume (initially cytotoxic with a secondary shift to vasogenic edema and gliosis). Notably, delayed edema plus gliosis appeared also after decompression even without preceding trauma.

**Conclusion:**

In prolonged outcomes, craniectomy applied after closed head injury in mice aggravates posttraumatic brain edema, leading to additional functional impairment. This effect is, however, transient. Treatment options that reduce brain swelling after decompression may accelerate neurological recovery and should be explored in future experiments.

## Introduction

The aim of decompressive craniectomy (DC) is to reduce intracranial pressure (ICP) by removing part of the skull (1). DC may be performed for a variety of indications (1, 2), and traumatic brain injury (TBI) remains the flagship condition for which neurosurgeons are employing DC (3, 4), usually due to posttraumatic brain edema (5–8).

The positive effects of skull decompression in TBI are not exclusively mechanistic. In addition to reducing ICP (5, 9–11) and optimizing intracranial compliance (12, 13), DC is capable of improving cerebral blood flow, cerebrovascular reactivity, brain tissue oxygenation and cerebral metabolism (3, 5, 14, 15). However, the clinical data on functional recovery after TBI and DC remain controversial. The results of randomized clinical trials (DECRA and RESCUEicp) are not equvocal: DECRA demonstrated that DC increases the risk of an unfavorable posttraumatic course (16), while RESCUEicp reported improved recovery due to craniectomy (17). Importantly, RESCUEicp as well as some smaller studies demonstrated that the positive effect of DC increases over time (3, 17, 18).

This inconsistency in results of major clinical trials on DC may be only partially explained by differences (regarding the protocols and the population of patients) between both studies (DECRA vs. RESCUEicp). Therefore, a detailed analysis of the course of pathophysiological changes after TBI / DC in controlled setting of the translational animal model remains a valid research option. However, translating the dynamic effects of posttraumatic craniectomy into animal experiments remains a challenging task (19–24). In previous experiments, we were able to establish a mouse model of DC based on the closed head injury (CHI) paradigm (25, 26). In short-term observations, decompression performed early after CHI increased contusional blossoming, promoted brain edema formation and aggravated functional impairment (27). Until now, temporal dynamics in our murine DC model were followed only in a short time period (up to 3d) (28). We hypothesize that the effects of craniectomy on trauma sequelae will change their intensity over the prolonged course in animal experiments, as they do in clinical settings. To address this issue, we performed a series of experiments, observing neurological impairment and brain edema evolution at remote time points.

## Methods

### Animals and trauma model

The study was planned and performed according to ARRIVE guidelines (29) and in line with the laws for animal protection, including Directive 2010/63/EU, after approval of the local ethical board (17/2013, Saarland Ethical Commission).

Application of trauma according to the closed head injury (CHI) paradigm and microsurgical decompression of the left cerebral hemisphere was performed in male wild-type, CD-1 mice (9–12 weeks old, Charles River Laboratories) as described previously (for a detailed animal handling protocol and detailed description of the surgical procedure, see Supplementary material S1). Animals were randomly assigned to one of the following experimental groups: 1. sham-operated (sham); 2. decompressive craniectomy alone (DC); 3. closed head injury alone (CHI); 4. CHI followed by DC at 1 h post-TBI (CHI+DC) (*n* = 8 surviving animals in each group).

Surgical procedures were performed under isoflurane anesthesia and under monitoring of vital parameters, including body and head temperature. For groups CHI and CHI+DC, experimental TBI of moderate-to-severe degree was induced using a weight drop device [adapted from Chen et al. (25)]. Briefly, after inducing anesthesia and surgical exposure of the intact skull by skin incision, a 75 g weight was dropped from a height of 30 cm on a silicone cone resting on the exposed skull, resulting in focal brain injury to the left hemisphere. In the CHI+DC group, unilateral DC was performed 1 h after trauma as described previously (27): Here, after a midline longitudinal skin incision, the area of the skull over traumatized hemisphere was exposed. Thereafter, the temporal muscle was detached from its origin over the temporal bone, allowing additional exposition of temporal squama down to the floor of the middle fossa. Here, a margin of bone flap to be removed was outlined in the parietal and temporal bone using a dental drill (under constant cooling by the irrigation of the normal saline). Both parts of parietal and temporal bone were then removed down to the skull base and a dura opening over the hemisphere was created using microscissors and microforceps. Thereafter, the dura was left open, the muscle was freely readapted and the skin was closed using 5–0 polypropylene sutures. After hemostasis was achieved by temporary application of the gelatin foam, the skin was suture closed. In the DC group, the same procedure was performed (including incision and peeling off the dura parts) on the nontraumatized brain/skull 1 h following sham injury.

### Neurological assessment

The functional status of the animals was evaluated by an observer blinded to the treatment applied. The neurological severity score (NSS) (as the more complex neurobehavioral score) and beam balance score (BBS) (focused mostly on vestibular and motor function) were used (26, 30). For NSS, a 10-point scale was adapted from Stahel et al. (31). Animals were awarded one point for failure to perform a task. In the BBS, animals were awarded from 0 (good performance) to 5 points (not attempting to balance) during three attempts [adapted from Mikawa et al. (32)], and the mean was used for further analysis. The total balancing time (60 s each attempt = max 180 s) was analyzed separately.

NSS and beam balance performance, including BBS and beam balancing time (BBT), were assessed at the following time points: 1 day (24 h), 3 days (72 h), 7 days, 14 days and 28 days after the start of surgery.

### Magnetic resonance imaging

After functional assessment at each of the time points (1d, 3d, 7d, 14d and 28d), animals were subjected to magnetic resonance imaging (MRI), described and published in detail as an open-access protocol elsewhere (see Supplementary material 2 for detailed description) (33). In brief, MRI scanning was performed under isoflurane anesthesia with constant monitoring of vital parameters. MR sequences were acquired according to multislice multiecho (MSME, T1 weighted), turbo spin echo (TSE, T2 weighted) and echo planar imaging (DWI, diffusion weighted) paradigms.

Brain edema was identified in T2-weighted images and acquired diffusion coefficient (ADC) maps calculated from the DWI data, and matching regions of interest (ROI) were manually created with the Paravision 5.1 ROI tool. The resulting size measurements (in pixels and mm^2^) were exported, and the total volume of the lesions was calculated.

### Histological analysis

Twenty-eight days after surgical treatment, the animals were sacrificed using transcardial perfusion with buffered formaldehyde solution; the brains were removed, fixed, and paraffin embedded, and serial coronal sections of the brains (5 μm) were made, presenting ROIs, i.e., coronal slices displaying hippocampal areas CA1 and CA3, as assessed using the stereotactic mouse brain atlas (34). The neighboring sections were stained with H&E (hematoxylin and eosin) as well as by the Nissl staining technique and immunostained with anti-glial fibrillary acidic protein (anti-GFAP) antibody (as described in Supplementary material S3).

All sections were microscopically analyzed by an independent observer blinded to the treatment of the animal.

### Statistical analysis

For all time points (1d, 3d, 7d, 14d and 28d), the weight of the experimental animals (including Δ weight), neurological impairment according to the NSS and BBS, balancing time in the BB test (BBT) and volume of lesions were expressed as the mean ± SEM.

The Gaussian distribution was validated using the Shapiro–Wilk test. For the values with a non-Gaussian distribution and for the nonparametric values (NSS and BBS), the Kruskal–Wallis test followed by Dunn’s multiple comparison *post hoc* test was applied. For the parametric values with Gaussian distribution, one-way ANOVA with Bonferroni *post hoc* correction was used. For each animal, a global injury score (as demonstrated by Δ weight, NSS, BBS, BBT, volume of edema) was created by averaging the five respective measures at single time points (1d, 3d, 7d, 14d, 28d) and analyzed across the treatment groups using the Kruskal–Wallis test or one-way ANOVA.

For analysis of both treatment and time effects, two-way ANOVA (for parametric variables with Gaussian distribution) and Friedman’s two-way analysis of the variance by ranks (for nonparametric and/or non-Gaussian variables) were used, treating each mouse as a block and time as a factor (time and treatment as two independent factors). For all parts of the assessment, significance was set at *p* < 0.05.

## Results

### Perioperative management, mortality and morbidity

The early mortality (surgical part of the experiment, up to 3 h) was highest in the CHI+DC group (36%), followed by 33% in the CHI group, 21% in the DC group and 10% in the sham group. In the prolonged course, additional loss of experimental animals was observed, increasing total mortality numbers to 66% for the CHI+DC group, 45% in the CHI group, 53% in the DC group and 20% for sham animals.

### Weight of animals

**Figure 1:**
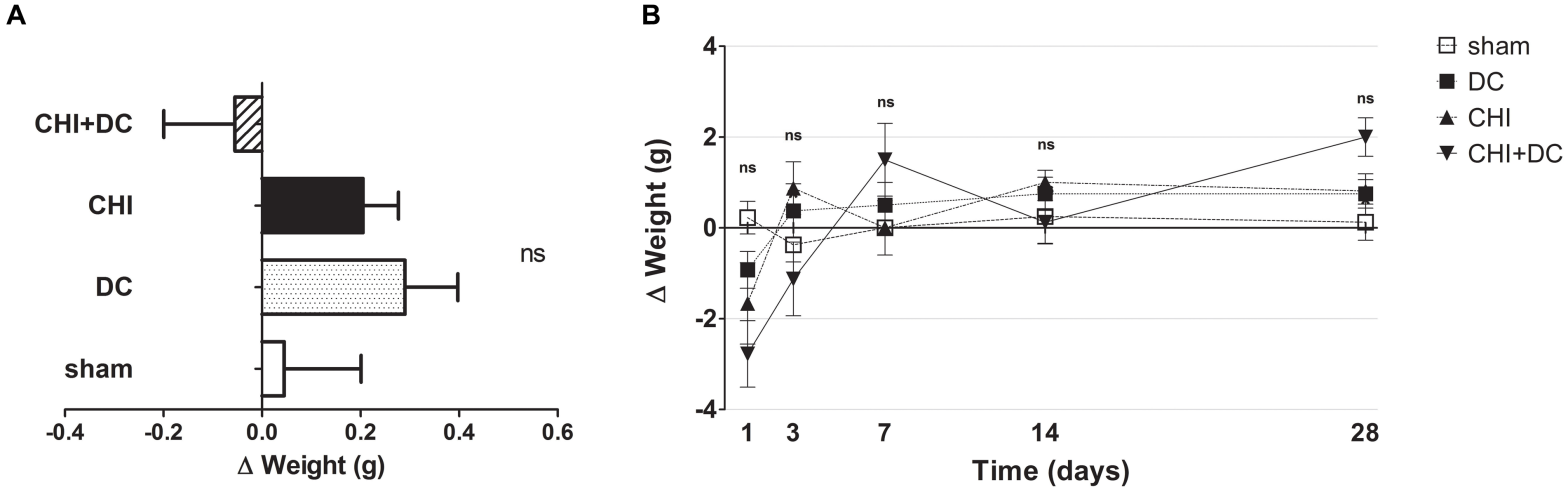
Histograms demonstrating weight loss (difference of two consecutive weightings as a more detailed parameter of well-being and recovery than plain weight assessment) after trauma/surgical treatment during the prolonged posttraumatic course (up to 28d). **(A)** The total weight loss score (obtained by averaging the weight change values assessed at separate time points) is presented. Animals treated with surgical decompression after trauma (CHI + DC) displayed a negative index, while the positive result of the calculation could be attributed to the rest of the groups, although no significant difference between the groups could be demonstrated. **(B)** Graph demonstrating the time course of weight loss/gain during the whole experiment. The most dynamic changes occurred during the first 7d of observation, with the CHI+DC group being affected by the most considerable weight loss compensated by its spectacular gain later on. However, none of the single time point comparisons revealed a significant difference between the groups.

No differences in initial weight were documented between the groups. Both the weight and its changes were strongly affected by the time course (weight: one-way analysis: *p* > 0.05, ns for all groups and all time points; two-way analysis: p > 0.05, ns for treatment effect; *p* < 0.0001,*** for time effect; Δ weight; two-way analysis: *p* = 0.22, ns for treatment effect; *p* < 0.0001,*** for time effect; Figures 1A,B).

### Functional outcome

**Figure 2:**
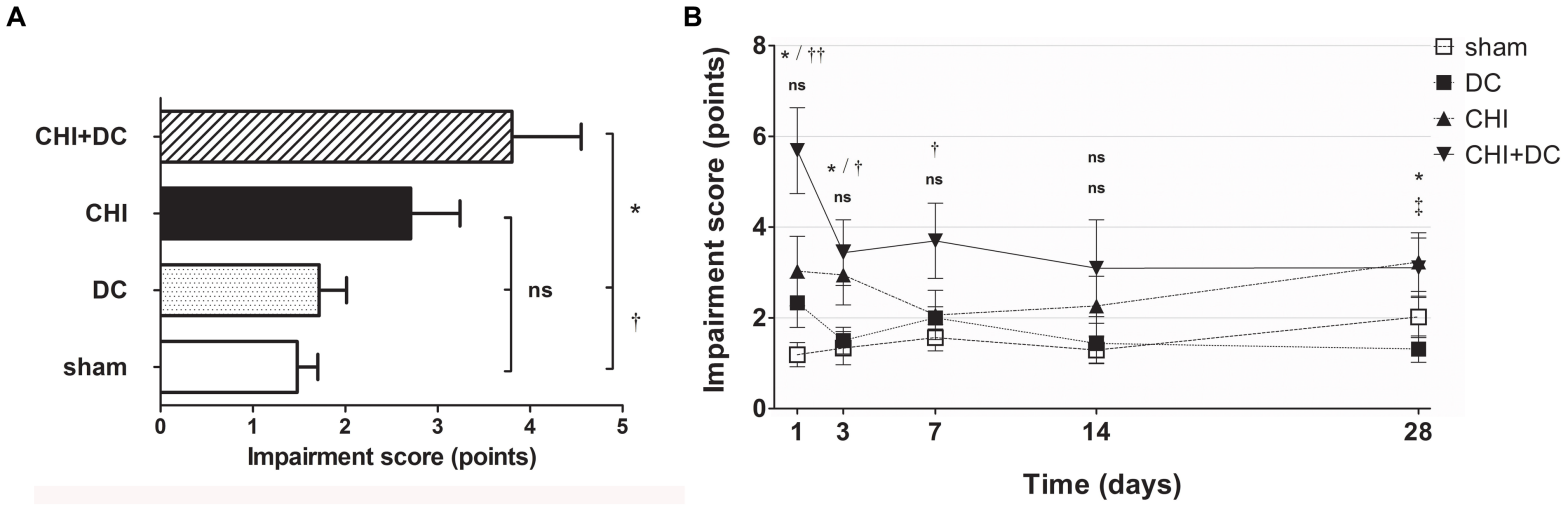
Histograms showing neurological impairment after trauma/sham injury according to the Neurological Severity Score (NSS) during the prolonged posttraumatic course (up to 28d). **(A)** The total impairment score (obtained by averaging the NSS values assessed at separate time points) is demonstrated. Animals treated with surgical decompression after trauma (CHI + DC) displayed the most profound impairment, which was significantly higher than that of the sham or decompression-only (DC) group (CHI+DC vs. DC, * *p* < 0.05; CHI+DC vs. sham, † *p* < 0.05). **(B)** Graph demonstrating the time course of neurological impairment and recovery based on NSS assessment. Note the high level of functional impairment among CHI+DC animals at 1d postinjury, concordant with our previous report (27) and successive improvement of the NSS values. The description of significant differences between the groups is double-noted; the upper index refers to the comparison between CHI+DC vs. DC (*) and CHI+DC vs. sham (†), and the lower index refers to the comparison between CHI vs. sham (§) and CHI vs. DC (‡). In the early course, there was a significant impairment of animals with posttraumatic decompression (1d CHI+DC vs. DC: *, *p* < 0.05; vs. sham: ††, *p* < 0.01; 3 d CHI+DC vs. DC: *, *p* < 0.05; CHI+DC vs. sham: †, *p* < 0.05; 7 d CHI+DC vs. sham: †, *p* < 0.05). The fluctuation of neurological recovery resulted in late impairment (28d CHI+DC vs. DC: *, *p* < 0.05). Notably, the trauma animals without craniectomy (CHI) also demonstrated delayed functional damage (28d, CHI vs. DC: ‡, *p* < 0.05).

According to NSS, animals subjected to craniectomy after trauma (CHI+DC) demonstrated the poorest global performance (Figure 2A), with the most profound impairment at the first two posttraumatic measurement points (1d and 3d after injury) and present to a lesser degree at remote time points (7d and 28d; Figure 2B). According to two-way analysis, time course significantly impacted functional recovery according to NSS (NSS; two-way analysis: *p* = 0.01,* for treatment effect; *p* = 0.0027,** for time effect).

**Figure 3:**
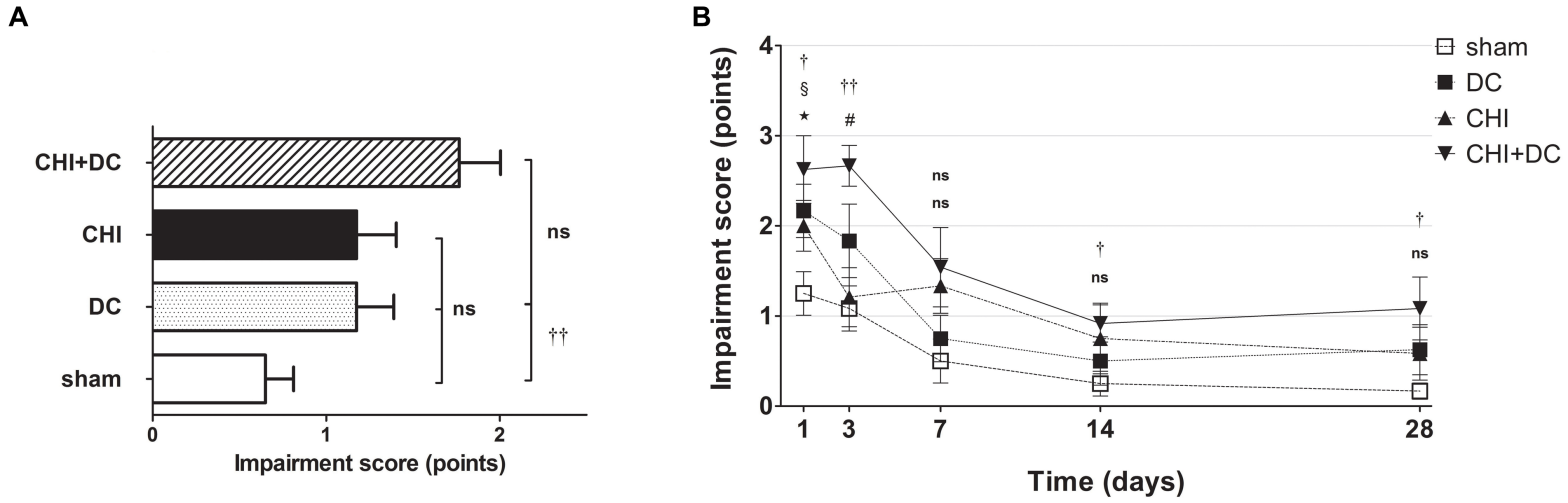
Histograms demonstrating neurological impairment after trauma/sham injury according to the Beam Balance Score (BBS) during the prolonged posttraumatic course (up to 28d). **(A)** The total impairment score (obtained by averaging the BBS values assessed at separate time points) is presented. Again, animals treated with surgical decompression after trauma (CHI + DC) had the poorest performance; however, the only significant difference was noted between this treatment group (CHI+DC) and the secondary control group (sham) (CHI+DC vs. sham: ††, *p* < 0.01). **(B)** Graph demonstrating the time course of neurological impairment and recovery based on BBS assessment. Similar to the NSS results, functional impairment was highest among CHI+DC animals in the early phase postinjury. The description of significant differences between the groups is double-noted; the upper index refers to the comparison between CHI+DC vs. DC (*) and CHI+DC vs. sham (†), the lower index refers to the comparison between CHI vs. sham (§) and CHI vs. DC (‡), and the additional symbols describe the significance of the difference between CHI+DC vs. CHI (#) and between DC vs. sham (). The most profound impairment of CHI+DC animals was observed in the early course, as this group demonstrated poorer performance than sham littermates (1d CHI+DC vs. sham: †, *p* < 0.05; 3d CHI+DC vs. sham: ††, *p* < 0.01). In the late course, successive improvement of neurological function in all groups was seen (as demonstrated by the downward slope of the impairment curves). However, the difference between sham-treated and CHI+DC animals remained significant (saved time point 7 d): (14 d CHI+DC vs. sham: †, *p* < 0.05; 28 d CHI+DC vs. sham: †, *p* < 0.05). In the case of CHI animals, initial impairment (1d CHI vs. sham: §, *p* < 0.05) and substantial improvement between 1d and 3d were documented, leading to a significant difference between this group and trauma + craniectomy animals (3d CHI+DC vs. CHI: #, *p* < 0.05) at the latter time point. Nevertheless, statistical significance between the CHI+DC group and the primary control (DC) was lacking (CHI+DC vs. DC: ns, *p* > 0.05 for all time points). This effect was probably due to the poor performance of decompression-only animals, which was significant at 1d postinjury (1d DC vs. sham: , *p* < 0.05).

A different pattern was seen in the analysis of BBS. Here, the average impairment score of CHI+DC animals was higher than that of sham but not DC animals (Figure 3A). In a detailed analysis of separate time points, at 1d postinjury, the trauma animals (CHI) and the DC group displayed clear deficits compared with the sham control. In CHI+DC animals, the balancing performance was poorer than that in the sham group but not the DC group. A similar pattern could be demonstrated at 3d, 14d and 28d postinjury. In contrast, in the prolonged outcome group (7d-28d), the BBS performance of the CHI group did not differ from that of the sham and DC reference groups (Figure 3B). Both treatment and time affected the beam balance performance results (BBS; two-way analysis: *p* = 0.0097,** for treatment effect; *p* < 0.0001,*** for time effect).

**Figure 4:**
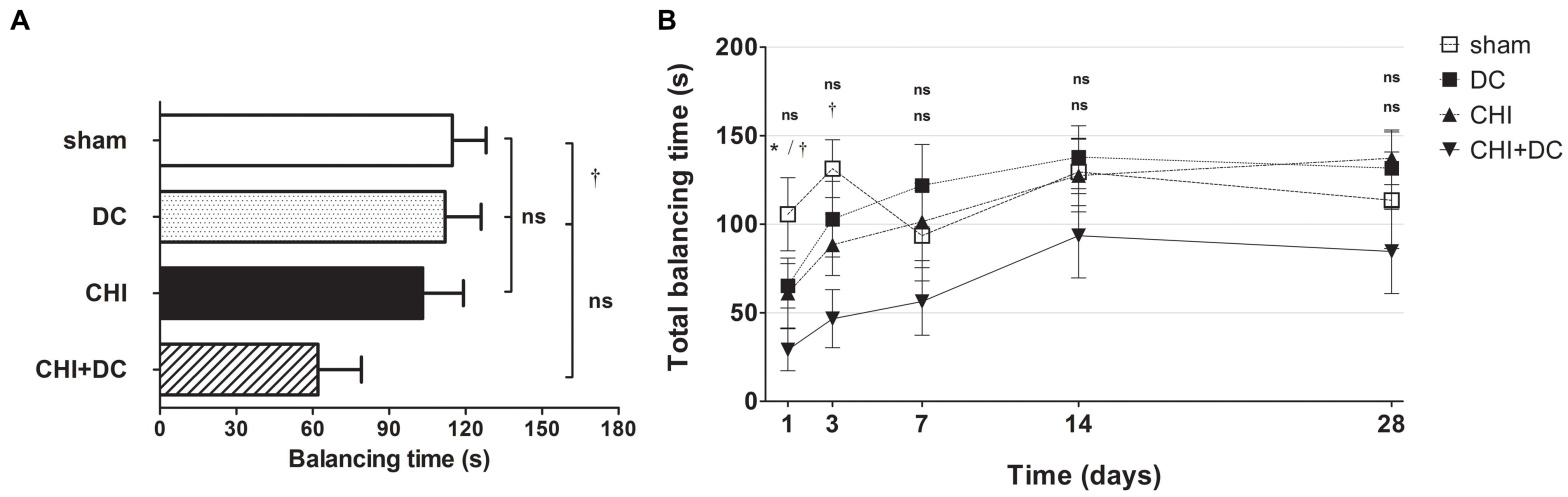
Histograms demonstrating neurological performance after trauma/sham injury according to the total balancing time in the beam balance test (BBT) during the prolonged posttraumatic course (up to 28d). Note the reversed order of groups in both parts of the figure, as the lowest scores in this **(B)** represent the most profound impairment. **(A)** The total impairment score (obtained by averaging the BBT values assessed at separate time points) is presented. Similar to the results of the NSS and BBS assessments, animals treated with surgical decompression after trauma (CHI + DC) had the poorest performance (i.e., the shortest balancing times). Similar to the BBS results, the difference between the groups was observed only between this treatment group (CHI+DC) and sham animals (CHI+DC vs. sham: †, *p* < 0.05). **(B)** Graph demonstrating the time course of neurological impairment and recovery based on BBT assessment. Again, functional impairment was highest among CHI+DC animals in the early phase postinjury. As observed previously, the most profound difference could be noted at 1d postinjury; here, both primary and secondary controls (DC and sham animals, respectively) demonstrated significantly longer balancing times than the trauma + decompression group (1d CHI+DC vs. DC: *, *p* < 0.05; vs. sham: †, *p* < 0.05). This effect could also be demonstrated at 3d after trauma in regard to the sham group (3d CHI+DC vs. sham: †, *p* < 0.05). Later, all but the sham group (with an undulating course of BBT performance score) demonstrated successive improvement with an increase in balancing times. Nevertheless, for prolonged outcomes (7d-28d), the differences between the groups were not statistically significant.

The analysis of averaged beam balancing time (BBT) mirrored the BBS results. Here, the average BBT of CHI+DC animals was the lowest among the groups and was significantly lower than that in the sham group but not the DC group (Figure 4A). At separate time points, BBT demonstrated the impairment of CHI+DC animals, which was significant at 1d and at 3d postinjury. At the same time points, no significant difference in balancing time could be stated between CHI animals and reference groups. At more remote time points (7d-28d), no significant differences could be discerned (Figure 4B). In two-way analysis, the effect of time but not of treatment on BBT was significant (BBT; two-way analysis: *p* = 0.07, ns for treatment effect; *p* < 0.0001, *** for time effect).

### Radiological assessment

**Figure 5:**
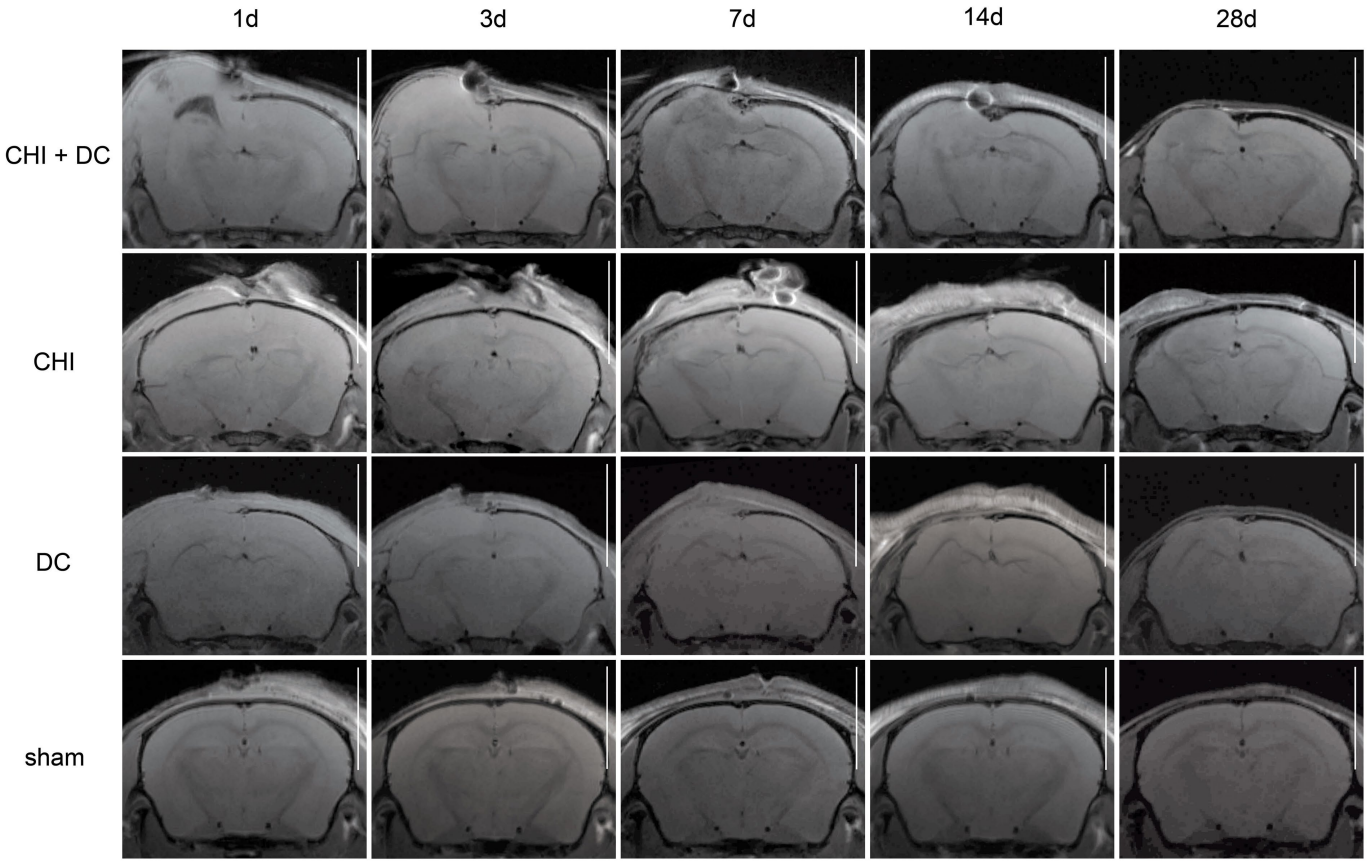
Panels of images representing the time course of radiological changes after trauma/decompressive craniectomy: time course (horizontal) vs. different treatment groups (vertical). Here, the MRI scans as T1-weighted images are presented. See description and interpretation of radiological changes in the main text. The scans were obtained in representative animals (each row demonstrates the radiological course in the same animal) using a 9.4 Tesla MRI scanner; white bar = 5 mm.

**Figure 6:**
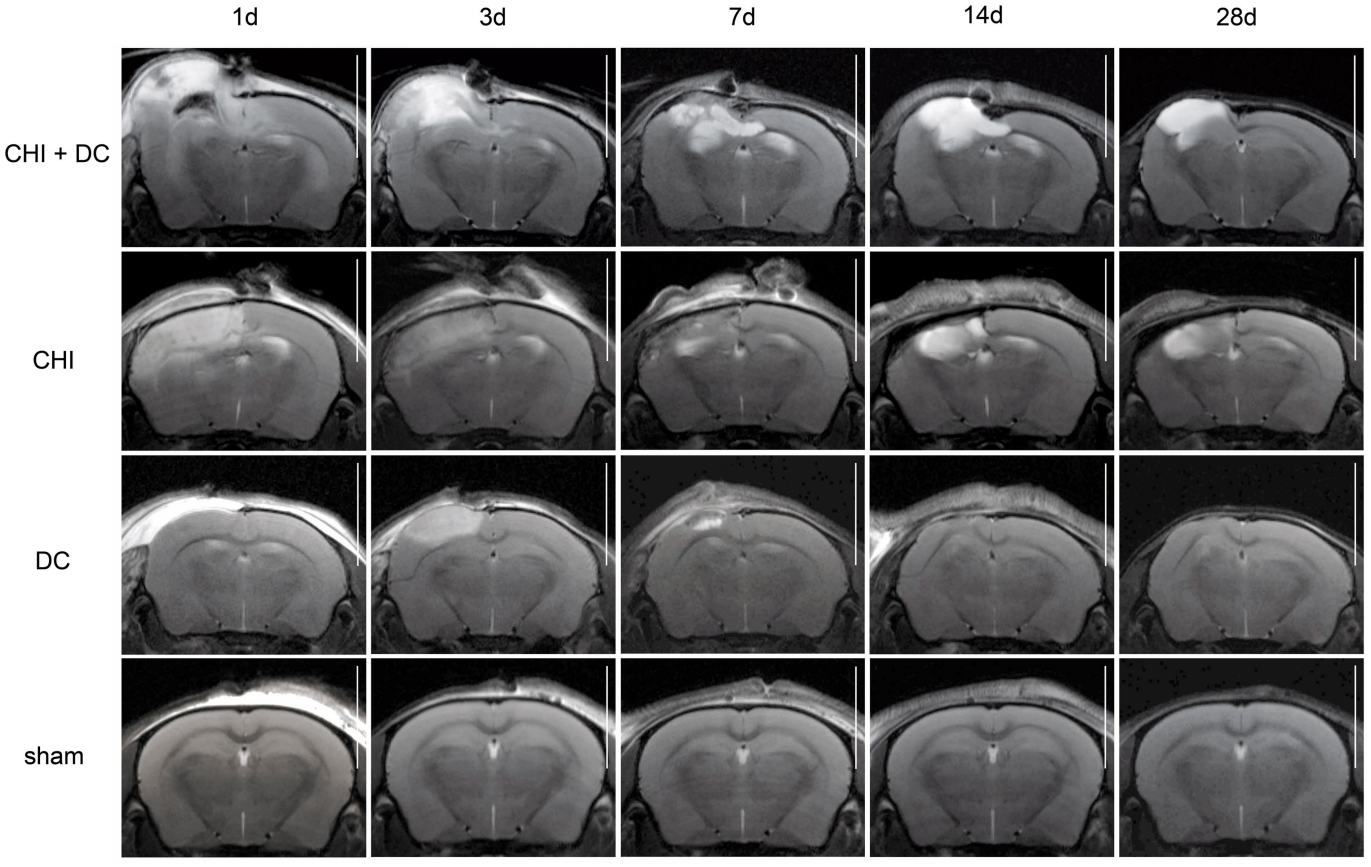
Set of images similar to Figure 5, including T2-MRI scans. See description and interpretation of radiological changes in the main text. Again, the scans were obtained in representative animals (each row demonstrates the radiological course in the same animal) using a 9.4 Tesla MRI scanner; white bar = 5 mm.

**Figure 7:**
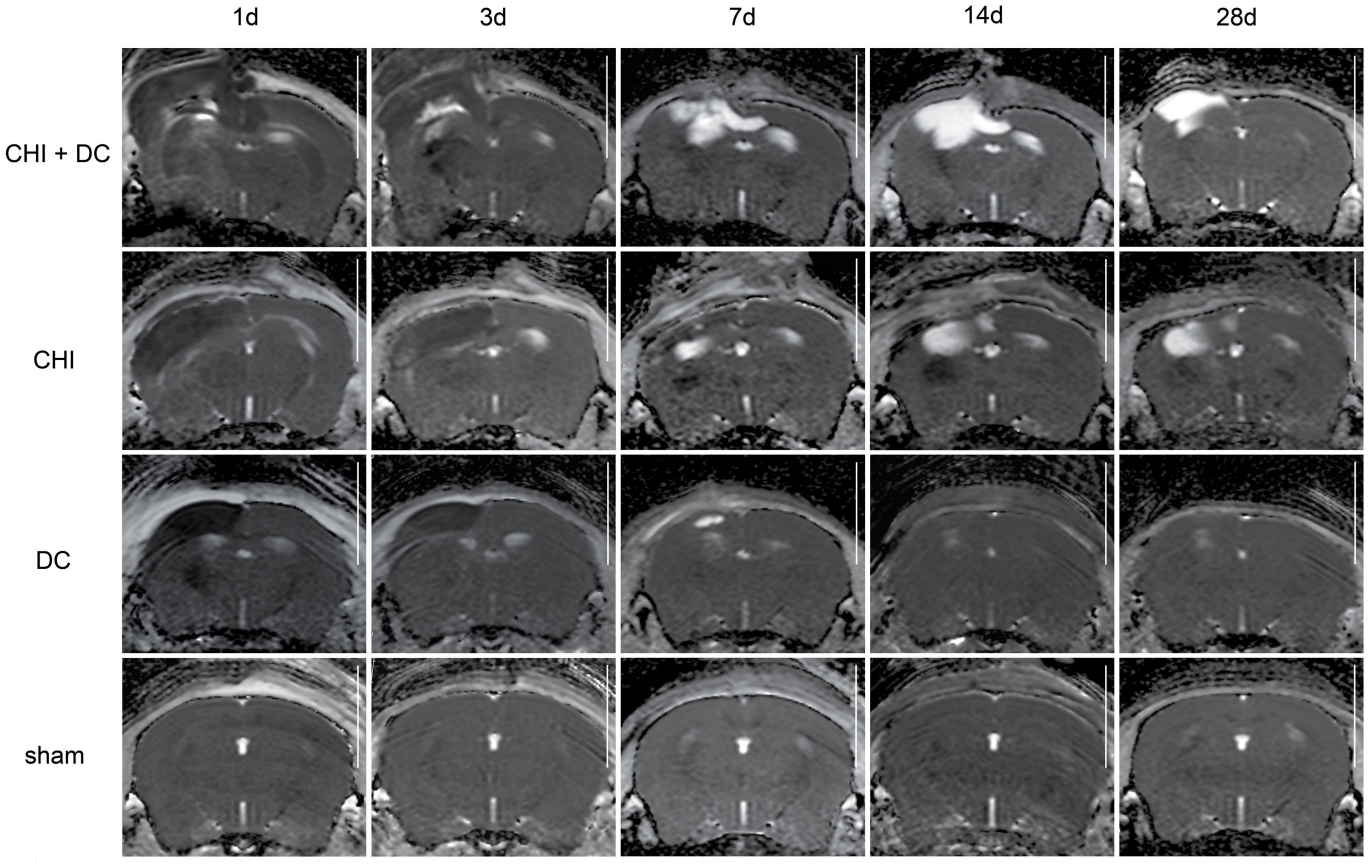
Set of images similar to Figures 5 6, presenting ADC-MRI maps. See description and interpretation of radiological changes in the main text. Again, the scans were obtained in representative animals (each row demonstrates the radiological course in the same animal) using a 9.4 Tesla MRI scanner; white bar = 5 mm.

The MRI-radiological features after different treatment combinations are presented in Figures 5–7.

As expected, sham animals demonstrated no discernible brain damage (including no brain edema formation). During the postoperative course, only proper healing of the superficial scalp wound could be demonstrated.

In contrast, the DC group demonstrated slight deformation of the cortex areas underlying the skull window as early as 1d after surgery. Initially, only the superficial cortex layer demonstrated some hyperintensity in T2-scans. However, at this time point, a restriction of fluid diffusion (suggestive of cytotoxic edema) that reached more profound areas of the cortex could be displayed in ADC images. These changes faded over time point 3d and were accompanied by appearance of smaller, ADC-hyperintense areas that resolved at time point 14d up to 28d. However, some small ADC- (and, to lesser degree, T2-) hyperintense cortical and subcortical changes remained visible up to endpoint 28d, resulting in deformation of the region underlying the craniectomy. This could be visualized in T1 images as some distortion of anatomical structures (cortical areas, corpus callosum and hippocampal area). Notably, no signs of subgaleal CSF collection or changes specific for brain tissue infection could be noted in the DC group.

In the CHI group, the features of brain edema formation and parenchymal changes, typical for a murine CHI model, were displayed. Thus, an early peak of cerebral swelling (at 1d and 3d) could be demonstrated. Here, the area of increased water content was hyperintense in the T2 scan but hypointense in the ADC-weighted images, suggesting the cytotoxic character of edema. The accompanying midline shift indicated the space-occupying effect of edema. In the further course, the changes evolved to the form of ADC-mixed lesions with an increasing hyperintense component, most prominent 28d after injury.

The animals subjected to both trauma and decompression (CHI+DC) demonstrated the most striking injury pattern. Starting at the earliest time point (1d postinjury), massive swelling of the decompressed cortex, including external herniation of the cerebral tissue over the bone margin, was present. According to ADC mapping, the edema of the cortex was mostly of cytotoxic character (similar to the trauma-only group). However, at early stages, some ADC-hyperintense zones could be delineated in subcortical areas (from 1d up to 7d). Starting at 7d, the extent of external herniation subsided, and the diffuse edematous zones (initially hypointense in the ADC map) evolved into T2- and ADC-hyperintense areas, with the signal intensity increasing during the protracted course. At the same time, the subcortical hyperintense areas expanded, peaking in size and intensity at 14d with a subsequent decline toward the endpoint (28d).

**Figure 8:**
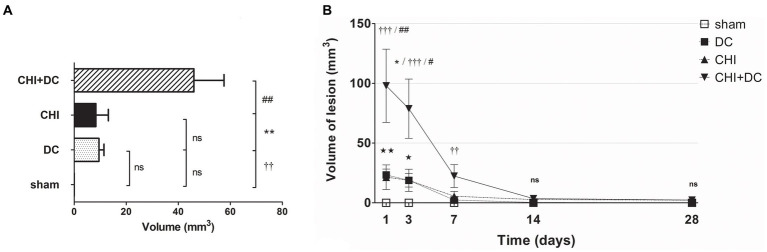
Histograms demonstrating volumetric analysis of MRI scans assessing the amount of lesion that is hypointense in ADC mapping (thus suggesting the presence of cytotoxic edema) during the prolonged posttraumatic course (up to 28d). **(A)** The averaged volume of hypointense lesions (obtained by averaging the volumetric data assessed at separate time points) is demonstrated. This type of structural change was most prominent in animals treated with surgical decompression after trauma (CHI + DC), as evidenced by a significant difference compared to all other groups (CHI+DC vs. DC, ** *p* < 0.01; CHI+DC vs. sham, †† *p* < 0.01, CHI+DC vs. CHI, ## *p* < 0.01). **(B)** Graph demonstrating the time course of ADC hypointense changes. The description of significant differences between the groups is single-noted, referring to comparisons between CHI+DC vs. DC (*), CHI+DC vs. sham (†) and CHI+DC vs. CHI (#); the additional symbols describe the significance of differences between DC vs. sham (). Obviously, the animals treated by posttraumatic decompression displayed the highest volume of the lesion, with the early peak at the initial time point of 1d. The difference from other groups (in particular to the secondary reference, i.e., sham animals) remained significant up to 7d postinjury (1d CHI+DC vs. sham: †††, *p* < 0.001; vs. CHI: ## *p* < 0.01; 3 d CHI+DC vs. DC: *, *p* < 0.05; CHI+DC vs. sham: †††, *p* < 0.001; vs. CHI: # *p* < 0.05; 7 d CHI+DC vs. sham: ††, *p* < 0.01). Notably, “true sham” animals subjected to decompression but not to trauma also displayed a significant amount of hypointense changes (1d DC vs. sham: 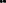, *p* < 0.01; 3d DC vs. sham: , *p* < 0.05). In all animals, this type of lesion faded with the time course, with no meaningful volume at 14d and 28d.

In volumetric analysis, sham-treated animals did not present any discernible lesions, while CHI+DC animals demonstrated the highest volume of lesions, particularly at early time points (1d to 7d; Figures 8A,B). Notably, at early time points (1d-3d), the craniectomy-only group (DC) (CHI) also demonstrated a significant amount of ADC hypointense changes.

**Figure 9:**
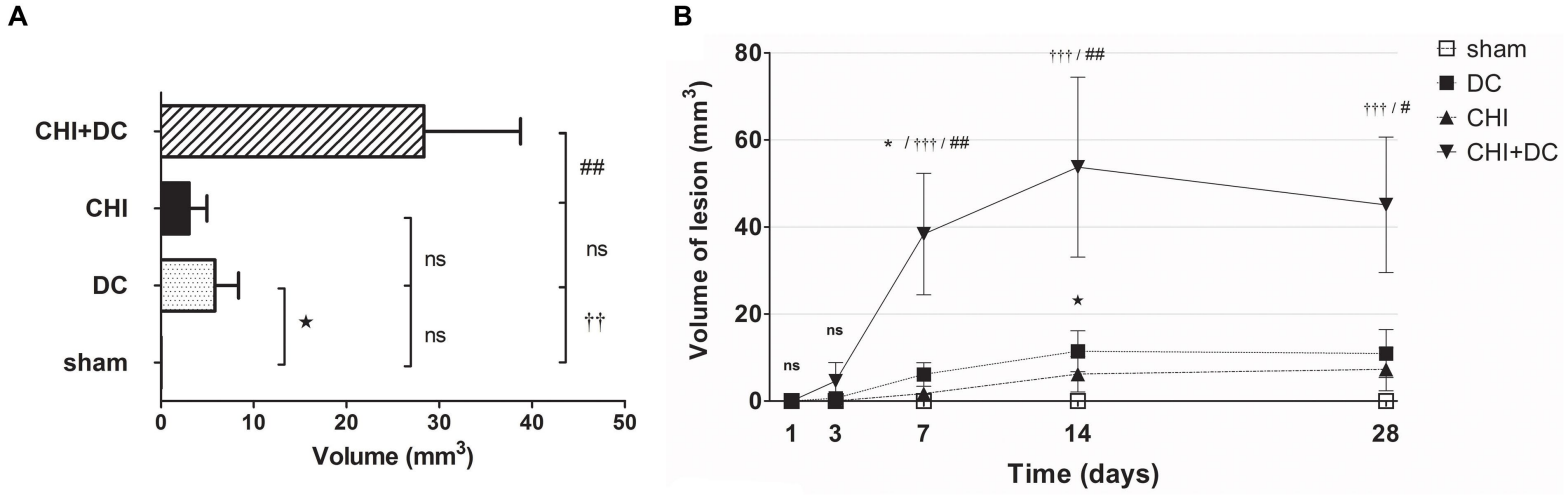
Histograms demonstrating volumetric analysis of MRI scans assessing the amount of lesion that is hyperintense in ADC mapping (thus suggesting presence of vasogenic edema, gliosis or both) during the prolonged posttraumatic course (up to 28d). **(A)** The averaged volume of hyperintense lesions (obtained by averaging the volumetric data assessed at separate time points) is presented. Similar to hypointense changes, the average lesion volume was most prominent in animals treated with surgical decompression after trauma (CHI + DC). This difference was statistically significant when compared with secondary reference (sham) as well as with trauma-only animals (CHI+DC vs. sham, ††, *p* < 0.01, CHI+DC vs. CHI, ##, *p* < 0.01) but not with primary reference (DC) animals (CHI+DC vs. DC, ns, *p* > 0.05). This observation presumably relies on a highly significant level of structural changes in the DC group itself (DC vs. sham: , *p* < 0.05). **(B)** Graph demonstrating the time course of ADC-hyperintense changes. The description of significant differences between the groups is single-noted, referring to comparisons between CHI+DC vs. DC (*), CHI+DC vs. sham (†) and CHI+DC vs. CHI (#); the additional symbols describe the significance of differences between DC vs. sham (). The shape of the curves clearly demonstrates that this kind of lesion is a late phenomenon, with its peak at 14d post-trauma. Again, the highest amount of lesion was attributed to animals treated by posttraumatic decompression. The difference from other groups (in particular to the secondary reference, i.e., sham animals) became significant starting 7d postinjury (7d CHI+DC vs. sham: †††, *p* < 0.001; vs. CHI: ## *p* < 0.01; 14d CHI+DC vs. sham: †††, *p* < 0.001; vs. CHI: ## *p* < 0.01; 28d CHI+DC vs. sham: †††, *p* < 0.001; vs. CHI: # *p* < 0.05). However, the difference from the primary reference (DC animals) became significant only at 7d after surgery (7d CHI+DC vs. DC: *, *p* < 0.05), probably due to the meaningful amount of hyperintense changes in the DC group thereafter, reaching a plateau at 14d post-surgery (14d DC vs. sham: , *p* < 0.05).

Regarding hyperintense changes, the CHI+DC group again demonstrated the highest magnitude of lesions. However, the craniectomy-only group also demonstrated significant lesions (Figure 9A). These changes also displayed a different progression course, with their appearance and peak starting at 7 days. As an ephemeral observation, the DC group demonstrated a significant volume of hyperintense changes at 14d (Figure 9B).

**Figure 10:**
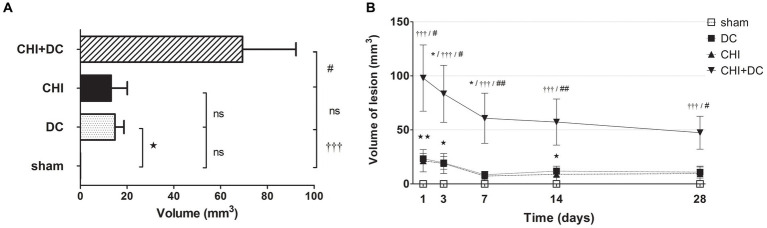
Pair of histograms demonstrating the total volume of lesions (both hypo- and hyperintense in ADC mapping) during the prolonged posttraumatic course (up to 28d). **(A)** The averaged total lesion volume (obtained by averaging the volumetric data assessed at separate time points) follows the pattern displayed by hyperintense-only changes. Thus, the total edema volume was highest in animals treated with surgical decompression after trauma (CHI + DC), and again, this difference was statistically significant when compared with secondary reference (sham) as well as with trauma-only animals (CHI+DC vs. sham, ††, *p* < 0.01, CHI+DC vs. CHI, ##, *p* < 0.01) but not with primary reference (DC) animals (CHI+DC vs. DC, ns, *p* > 0.05). After summation of both lesion types, a significant level of structural changes in the DC group was reached (DC vs. sham: , *p* < 0.05). **(B)** Graph demonstrating the time course of total lesion volume. The description of significant differences between the groups is single-noted, referring to comparisons between CHI+DC vs. DC (*), CHI+DC vs. sham (†) and CHI+DC vs. CHI (#); the additional symbols describe the significance of differences between DC vs. sham (). The time course resembles that of hypointense changes, with an early peak immediately after trauma/surgery and a plateau starting at +/− 7d postinjury. In this part of the analysis, the trauma + craniectomy group demonstrated a total lesion volume that was significantly higher at all time points compared with both sham animals and the trauma-only group (1d CHI+DC vs. sham: †††, *p* < 0.001; vs. CHI: # *p* < 0.05; 3d CHI+DC vs. sham: †††, *p* < 0.001; vs. CHI: # *p* < 0.05; 7d CHI+DC vs. sham: †††, *p* < 0.001; vs. CHI: ## *p* < 0.01, 14d CHI+DC vs. sham: †††, *p* < 0.001; vs. CHI: ## *p* < 0.01; 28d CHI+DC vs. sham: †††, *p* < 0.001; vs. CHI: # *p* < 0.05). Regarding the primary reference (DCs), significance was reached only at 3d and 7d postinjury (3d CHI+DCs vs. DCs: *, *p* < 0.05; 7ad CHI+DCs vs. DCs: *, *p* < 0.05). Most importantly, this pattern results from a considerable amount of total lesion, seen in the DC group, as evidenced by significant differences at time points 1d, 3d and 14d (1 d DC vs. sham: 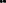, *p* < 0.01; 3 d DC vs. sham: , *p* < 0.05; 14 d DC vs. sham: , *p* < 0.05).

Similar results were obtained in the calculation, where both types of lesion (hypo- and hyperintense in ADC assessment) were added and analyzed as one variable. Obviously, the total lesion volume after averaging was highest in the CHI+DC group. Additionally, in the craniectomy-only group, the sum of lesion volume was higher than that in the sham group (Figure 10A). On the time axis, profound damage was observed in CHI+DC animals at all time points assessed. The DC group displayed a significant amount of total lesion at 1d, 3d and 14d after surgery (Figure 10B).

### Histological analysis

**Figure 11:**
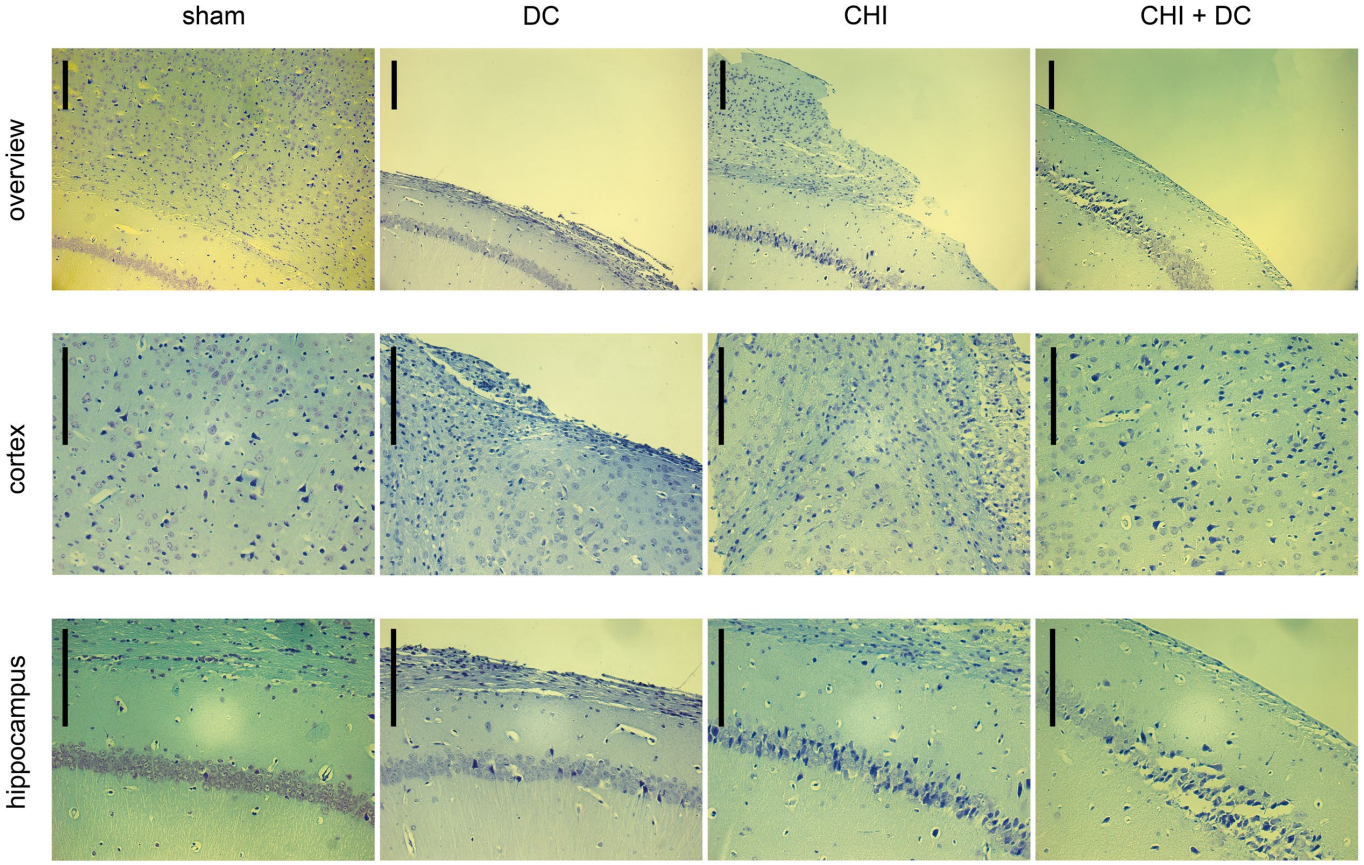
Photomicrographs of coronal paraffin sections obtained from animals subjected to sham treatment, decompressive craniectomy (DC), closed head injury (CHI), or closed head injury followed by DC (CHI + DC). All animals were sacrificed 28d postinjury. The sections were obtained from representative animals. Upper panel provides an overview of cortical layers (1-6b), subcortical structures (corpus callosum, alveus) and hippocampal CA1 area (striatum oriens, pyramidal layer and upper part of stratum radiatum), ipsilateral to injury site (magnification 100x, bar = 200 μm); middle row demonstrates cortex immediately adjacent the epicenter of injury (usually represented by defect of cortical brain tissue), layers 2/3 to 6b in greater magnification (200x, bar = 200 μm); bottom panel displays subcortical structures (corpus callosum and alveus) including stratum oriens, pyramidal layer and stratum radiatum of hippocampus (CA1) in greater magnification (200x, bar = 200 μm). Description of findings in the main text; note the necrotic area, mostly prominent in CHI+DC animal.

Two staining methods, Nissl staining to visualize neuronal loss and GFAP immunohistochemistry to depict glial activation, were implemented. These results are demonstrated in Figures 11 12.

**Figure 12:**
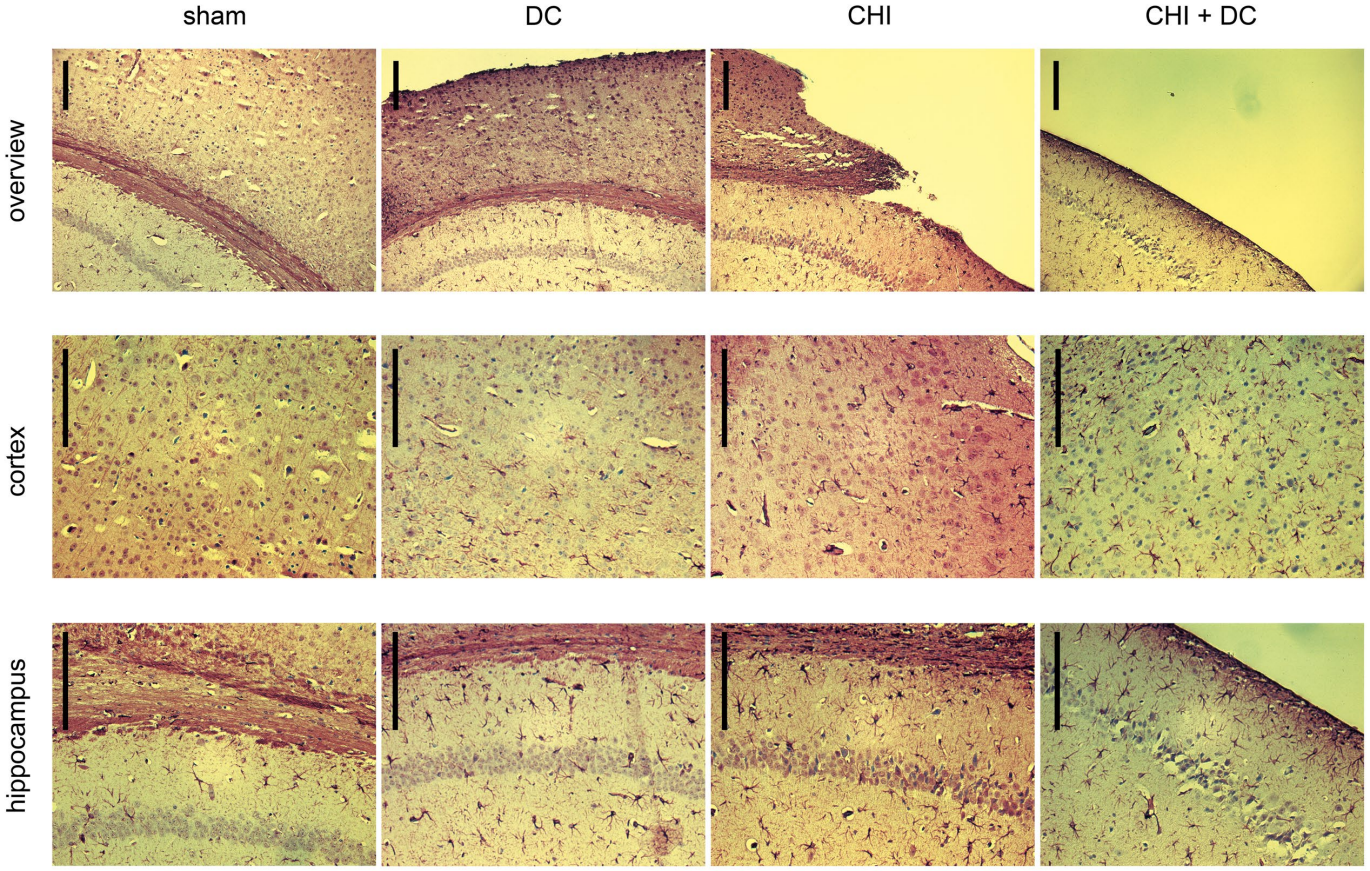
Photomicrographs of coronal paraffin sections obtained from animals subjected to sham treatment, decompressive craniectomy (DC), closed head injury (CHI), or closed head injury followed by DC (CHI + DC) and immunostained for GFAP. All animals were sacrificed 28d postinjury. The sections were obtained from representative animals. Similar to Figure 11, upper panel provides an overview of cortical layers (1-6b), subcortical structures (corpus callosum, alveus) and hippocampal CA1 area (striatum oriens, pyramidal layer and upper part of stratum radiatum), ipsilateral to injury site (magnification 100x, bar = 200 μm); middle row demonstrates cortex immediately adjacent the epicenter of injury (usually represented by defect of cortical brain tissue), layers 2/3 to 6b in greater magnification (200x, bar = 200 μm); bottom panel displays subcortical structures (corpus callosum and alveus) including stratum oriens, pyramidal layer and stratum radiatum of hippocampus (CA1) in greater magnification (200x, bar = 200 μm), description of findings in the main text.

According to Nissl staining (Figure 11), no changes in neuronal density or structure could be discerned in sham-treated animals. In contrast, the craniectomy performed without trauma (DC group) led to profound thinning of cortical layers with accumulation of several pyknotic neuronal nuclei in the area adjacent to the cranial window. However, the changes in subcortical zones were rather scarce, as represented by a couple of apoptotic neurons in the pyramidal layer of the hippocampus. In contrast, in CHI animals, a dramatic increase in the number of degenerating neurons could be demonstrated both in cerebral layers and, particularly, in the hippocampal pyramidal area of CA1. Animals subjected to both trauma and craniectomy (CHI+DC) displayed the most profound changes: not only total cortical depletion at the injury epicenter but also a dramatic reduction in the population of viable neurons (in favor of degenerating nerve cells) could be observed in the adjacent cortex. Additionally, the hippocampus demonstrated substantial neuronal loss, including not only apoptotic nerve cells but also several ghost neurons, and evidence of ongoing necrotic processes could be observed.

In GFAP staining (Figure 12), sham animals demonstrated no presence of activated astroglia across cortical layers and several GFAP-positive astroglia cells in CA1 hippocampal areas, consistent with previous anatomic descriptions (35).

As to the results of GFAP staining: Decompression performed without previous trauma led to the activation of numerous cortical astroglia and a noticeable increase in the population of GFAP-positive glial cells in the hippocampus (DC; here, for better visualization of cortical glial cells, an example of an animal with a less atrophic cortex was chosen). A similar pattern was observed among animals subjected to trauma, with even more accentuated astroglia-related hippocampal changes (CHI). In trauma + craniectomy animals, the most considerable increase in the population of activated astroglia can be recognized in the cortex adjacent to its atrophied part (although the hippocampus demonstrates a substantial increase in astroglia density as well).

## Discussion

In a previous series of experiments, we described the impact of craniectomy on early brain edema formation and on the short-term outcome (24 h) (27). Using the same paradigm, we expanded the follow-up timeframe to 28 days. We demonstrated a prolonged course of neurological impairment with partial recovery over time in animals with and without craniectomy. We also characterized the pattern of structural changes (gliosis and brain edema) following trauma and decompression using repetitive MRI scanning and endpoint histopathological analysis. Finally, we demonstrated the delayed negative effects of craniectomy, even without trauma.

In the past, Zweckberger et al. reported improved brain edema formation at 24 h after CCI in mice, resulting in reduced neurological impairment in the posttraumatic course (8d) (20) if the craniectomy was performed early enough (up to 3 h post-TBI) (21). In prolonged observation (28d), Friess et al. demonstrated reduced axonal damage, diminished white matter atrophy and less severe hippocampal neuronal loss in mice subjected to CCI without subsequent closure of skull defects (36). In a rat model, surgical decompression after FPI led to reduced brain edema formation with diminished AQP4 expression 48 h later (22). In CCI-treated rats, a positive effect of decompression (reduced volume of edema and contusion due to improved metabolic profile of the brain) was reported by Tian et al. up to 7d after trauma (23). Using the same model, Hou et al. reported improvement of behavioral changes by decompression, probably due to reduced impairment of synaptic function (24).

Our results are different from previous reports. Previously, using the CHI-DC model, we observed increased water content 6 h postinjury and augmented brain edema formation 24 h after TBI, accompanied by impaired neurological function (27, 37) in mice subjected to decompression (38). These characteristics bring our concept closer to early works on craniectomy performed by Cooper et al. using a cold lesion model. A 7-fold increase in brain water content was observed if craniectomy was performed during the phase of brain edema development (39). Notably, this early observation and our conclusions are not far from clinical practice. Additional brain swelling may be noted in 25 to 51% of TBI patients after craniectomy (40–45), occasionally leading to severe displacement of brain tissue outside the skull boundaries (known as external herniation or brain fungation) (46, 47). Certainly, a small decompression size increases the risk of this phenomenon (48, 49). However, external herniation may occur even if the craniectomy is properly sized (50). This effect was demonstrated in our experiment, along with a gradual decline in brain tissue prolapse over time. Thus, our results are consistent with some clinical reports (51) underlining the translational potential of our model.

The main goal of the current study was to analyze the dynamics of posttraumatic changes. Both trauma and surgical decompression trigger a long-lasting change in the physiological steady state (3, 18, 52). On the one hand, DC offers several benefits: improved intracranial compliance (12, 13), increased brain perfusion (5, 53, 54) and optimized brain metabolism (14, 55–57). On the other hand, craniectomy carries the disadvantage of brain exposure to external barometric pressure (55, 58), decreased CSF flow (59) or mechanistic shearing forces affecting the brain tissue (40, 48, 49, 60). The patients’ outcome is the net effect of both positive and negative events over time. Thus, not only the intensity but also the duration and timing of the given DC action must be considered. The best example here is the craniectomy effect on the ICP. In the DECRA trial, craniectomy was extremely effective against intracranial hypertension. As a result, DC was able to shorten the length of stay in the ICU, but this early effect did not positively influence the long-term outcome (16).

One of the strengths of our study is the depiction of brain edema evolution in MRI imaging. Similar to Tsenter et al. and Onyszchuk et al., we demonstrated a peak of hypointense changes at 24 h and their secondary decline thereafter, with gradual appearance of hyperintensive lesions, reaching a plateau at 14d (61, 62). We assumed that initial changes represent cytotoxic edema (7, 8, 63–67). The hyperintensive abnormalities are more difficult to interpret. On the one hand, vasogenic edema was present in various animal models of TBI (8, 68–71), including CHI (61, 72–74), and may manifest as ADC-hyperintensive areas (7, 68, 69, 74–76). On the other hand, their persistence (accompanied by T2-signal increase) suggests their gliotic or cavitary character (77, 78). Our histological analysis supports the latter interpretation, since we demonstrated the activation of astroglia and neuronal depletion resulting in tissue loss. Importantly, glial scarring has been described in other long-term rodent neurotrauma studies (79–82) and correlates with injury severity and functional outcome (83–88). Accordingly, in our interpretation, we demonstrated an early peak in cytotoxic edema, a secondary increase in vasogenic brain swelling and, finally, glial scar formation accompanied by neuronal loss 28d after TBI.

Most important observations were made in CHI+DC animals. Here, we demonstrated a significant impairment that was most prominent during the early posttraumatic phase. This pattern of functional improvement suits well the recovery potential seen in animal models of traumatic (24), ischemic (89, 90), or hemorrhagic brain injury (91) followed by surgical decompression. However, the prolonged negative effects of DC are specific to our model. One aspect is the evolution of cerebral swelling. From the very early time points, a massive increase in brain edema, accompanied by external herniation, could be observed. The main feature was the high volume of vasogenic edema, occurring earlier and more intensively than could be expected in the CHI model (61, 74). Here, the idea of increased water permeation from cerebral vessels to the brain parenchyma needs to be recalled. After craniectomy, vasogenic edema is no longer hampered by a rigid skull and may propagate freely (39, 92, 93). This effect may be accentuated by craniectomy-related changes in the expression of water channel proteins, such as aquaporin-4 (22, 33). Another consequence of pressure relief is the detamponade of contusional bleeding. The effect of hemorrhage blossoming after craniectomy is well recognized (46, 47, 50, 94, 95) and has been attributed to neurological deterioration in our short-term experiments (27) and in an animal model of subarachnoid hemorrhage (21). Finally, the negative impact of craniectomy on functional outcome may rely on shear load at the decompression rim (96), occurring while the progressive swelling is forcing the brain out of the cranial cavity (48, 49, 97). The decompressed brain may also suffer from chronic strains related to its deformation or to increased motility of the cerebral mass (98). This effect is potentially aggravated by changes in the mechanical properties of injured tissue (82, 99). This list of negative craniectomy effects needs to be kept in mind when discussing the unfavorable outcomes in the subset of TBI patients represented in our study by the CHI+DC group. In a previous report, we documented a short-term edema surge and contusional blossoming after DC (27). Currently, we have described the time course of brain swelling after trauma and decompression. Based on these observations, we conclude that our experimental protocol favors the deleterious effects of decompression over the protective ones, differing in this matter from most animal craniectomy studies.

The postsurgical course in animals decompressed without prior TBI is of particular interest. After removing the skull covering, the brain is exposed to the caprices of external pressure (58). This potentially explains the brain tissue deformation (98). The negative effect of pressure changes has been recognized previously (100, 101) and may potentially justify some deleterious effects of craniectomy, including syndrome of the trephined (46, 102, 103). To mimic this effect, occurring in the treatment phase between decompressive craniectomy and skull reconstruction (cranioplasty) was one of the goals of our translational study. For this reason we have chosen the animal model, not requiring skull opening and subsequent closure for trauma application itself. Basen on this paradigm, we could make an observation that the procedure of decompression itself is not neutral. Even with the use of microsurgical techniques and meticulous prevention of thermal injury due to drilling, surgical procedures on the rodent skull are burdened by some negative effects, such as hemodynamic or metabolic depletion (104) and proinflammatory, morphologic and functional damage (105). The relevance of skull manipulation for the “true sham effect” has been postulated previously (105–107) and justifies the paradigm of using “sham decompressed” animals as a reference group in current and future series of experiments.

Our study is not free from limitations. Our data were provided in a limited number of subjects, as required by the general 3R principle (reduction-replacement-refinement) (108). Thus, the statistical power of our analysis is much less than that of multicenter randomized trials. Further, the established technique of surgical skull decompression in small rodent model is mimicking but not perfectly replicating the craniectomy performed in human patient. In particular the lacking step dura reconstruction after its incision (as usually performed in clinical condition) may theoretically exaggerate the impact of external pressure on the exposed brain. However, also in current clinical practice, the securing of dura by free overlay of tissue (or tissue substitute) is preferred over the traditional watertight closure (109).

The most important caveat regards the risk of overinterpretation of our data in the clinical context. Our results should not be directly extrapolated to humans, even if the concept of our experiment was translational. Our protocol refers to a very specific situation (primary decompression for severe, focal TBI), while TBI in the clinical setting is heterogeneous, and decompression is usually performed for secondary ICP increase (2, 110).

## Conclusion

The main purpose of the study was to assess whether prolonged sequelae of decompressive craniectomy can be reproduced in experimental TBI and whether the effects of DC are persistent or transient. We demonstrated that decompressive craniectomy may accelerate the development of posttraumatic brain edema (in particular in its vasogenic form). The impact of skull decompression was not permanent and resolved over time. Two messages are important for clinical practice: The dynamics of posttraumatic brain edema are dramatically altered by skull decompression. Here, the cases of rapid malignant brain swelling with external herniation after decompression may be explained. Second, decompressive craniectomy after head trauma is the most dramatic but potentially not final form of treatment. We ought to seek brain edema therapies supplementary to surgical decompression.

## Data availability statement

The raw data supporting the conclusions of this article will be made available by the authors, without undue reservation.

## Ethics statement

The animal study was approved by Saarland University Hospital/Saarland Ethical Commission. The study was conducted in accordance with the local legislation and institutional requirements.

## Author contributions

JS: Conceptualization, Data curation, Formal analysis, Funding acquisition, Investigation, Methodology, Project administration, Supervision, Validation, Visualization, Writing – original draft, Writing – review & editing. VH: Data curation, Formal analysis, Investigation, Methodology, Visualization, Writing – original draft, Writing – review & editing. EK: Formal analysis, Investigation, Methodology, Validation, Visualization, Data curation, Writing – review & editing. AM: Data curation, Formal analysis, Funding acquisition, Investigation, Methodology, Project administration, Software, Validation, Visualization, Writing – original draft, Writing – review & editing. LA: Data curation, Investigation, Validation, Visualization, Writing – review & editing. KS: Conceptualization, Formal analysis, Investigation, Methodology, Project administration, Resources, Supervision, Writing – review & editing. JO: Conceptualization, Funding acquisition, Project administration, Resources, Supervision, Validation, Writing – review & editing.
