# Supplementary material for: Prolonged course of brain edema and neurological recovery in a translational model of decompressive craniectomy after closed head injury in mice
Source: Front Neurol. 2023 Nov 20;14:1308683. doi: 10.3389/fneur.2023.1308683 (PMC10694459; doi:10.3389/fneur.2023.1308683)
Supplement: Supplementary file 1 [file Data_Sheet_1.pdf]

Prolonged course of brain edema and neurological recovery in a translational model of decompressive craniectomy after closed head injury in mice

SUPPLEMENTARY DATA

Supplementary material 1:

Animal Handling protocol

All animal experiments were planned according to ARRIVE guidelines[1] and performed with approval by the local ethical board in line with the laws for animal protection, including Directive 2010/63/EU, and by following all institutional and national guidelines for the care and use of laboratory animals.

Male wild-type, CD-1 mice of 9-12 weeks of age naïve to previous surgical or drug treatment weighing 38.2 ± 1.1 g were acquired from Charles River Laboratories South Germany and kept in the local Animal Facility of the Institute for Clinical and Experimental Surgery (housed in groups of two to three animals under controlled conditions: 12 h:12 h light-dark cycle, water and food provided ad libitum). After the accommodation phase (> 3 d), the animals were transferred to the Neurosurgical Laboratory. Here, before any manipulation, animals were randomly assigned to one of the four following experimental groups: 1. sham-operated (sham); 2. decompressive craniectomy alone (DC); 3. closed head injury alone (CHI); 4. CHI followed by DC at 1 h post-TBI (CHI+DC) (n = 8 animals suitable for final analysis in each group). The sample size was defined using *a priori* assessment based on the functional and radiological parameters of the previous short-term study[2] (see statistical analysis). Here, the sample size of n = 8 surviving animals with a complete data set was estimated as the minimum necessary to achieve the required statistical power in functional assessment. Based on previous reports implementing rodent models of severe TBI[3,4] as well as our own previous experience[5], a mortality of 50% for TBI groups (CHI and CHI+DC) and 20% for non-TBI groups (sham and DC) was assumed.

For the surgical part of the experiment, an isoflurane anesthesia protocol was established based on the recommendations of several Animal Welfare Agencies[6] and under the assent of the local representative of the Animal Welfare Board, Saarland University. According to the protocol, spontaneously breathing mice were kept under general anesthesia by isoflurane inhalation (Forane®, Baxter, administered via Isoflurane Vapor® 19.1 device, Dräger; initial dose 3% in 97% O_2, maintenance 0.8-1.5%, in 99.2%-98.7% O_2).

For the CHI and CHI+DC groups, experimental TBI was induced using a weight drop device (adapted from Chen et al.)[7]. Briefly, the animals were placed on a heating pad with an additional heat lamp used if necessary. Target core and head temperatures were measured by a rectal probe and a needle temperature probe placed in the right temporal muscle, respectively, and maintained at 37 ± 0.5°C during the whole experiment. Following a midline longitudinal head skin incision, the skull was exposed, and the head was placed manually on the base of the weight drop device (Laboratory Tools Workshop, Department of Pharmacology, School of Pharmacy, The Hebrew University of Jerusalem, Israel). A 75 g weight was dropped from a height of 30 cm on a silicone cone resting on the exposed skull, resulting in focal brain injury to the left hemisphere. For groups 1 and 2 (sham and decompressive craniectomy alone), the same procedure was performed without weight drop. In the

CHI+DC group, unilateral DC was performed 1 h after trauma as described previously[2]. In brief, a bone flap was created in the parietal and temporal bone using a microsurgical high-speed drill under continuous saline irrigation to avoid heat injury to the underlying dura/brain (maintaining the physiological brain temperature was additionally controlled via a temperature probe/heating lamp as described above). The thin bone layer together with the adherent dura was then excised using microforceps and microscissors. The temporal bone was then removed in the same manner down to the skull base. During bone and dura manipulation, utmost care was applied to avoid mechanical damage to the underlying cortex. Blood oozing from the craniectomy margin was controlled by temporary application of small pieces of gelatin foam (Gelita Tampon®, Braun Surgical S.A.) at the bone rim moistened with saline. After removing the hemostatic material and ensuring sufficient bleeding control, the skin was closed using 6-0 polypropylene sutures (Premilene®, Aesculap AG). In the DC group, the same procedure was performed on the nontraumatized brain/skull 1 h following sham injury.

To harmonize the study protocol with previous experiments[2,8,9], anesthesia was sustained for 3 h. During the experiment, the fluid loss was compensated via administration of a body-weight adapted volume of sterile 0.9% NaCl solution (20 mL/kg body weight = ~ 0.8 mL/animal i.p.). After 3 h, the temperature probe was removed, and anesthesia was withdrawn. Animals were put back into cages (single animal per cage) and allowed to recover in an environment with controlled room temperature. Thereafter, the mice were returned to the Animal Facility of the Institute for Clinical and Experimental Surgery.

References to Supplementary Material 1

1. Kilkenny C, Browne W, Cuthill IC, Emerson M, Altman DG. Animal Research: Reporting in vivo Experiments—The ARRIVE Guidelines. *Journal of Cerebral Blood Flow & Metabolism.* 2011;31(4):991-993.

2. Szczygielski J, Mautes AE, Muller A, et al. Decompressive Craniectomy Increases Brain Lesion Volume and Exacerbates Functional Impairment in Closed Head Injury in Mice. *Journal of neurotrauma.* 2016;33(1):122-131.

3. Schwarzbold ML, Rial D, De Bem T, et al. Effects of traumatic brain injury of different severities on emotional, cognitive, and oxidative stress-related parameters in mice. *Journal of neurotrauma.* 2010;27(10):1883-1893.

4. Flierl MA, Stahel PF, Beauchamp KM, Morgan SJ, Smith WR, Shohami E. Mouse closed head injury model induced by a weight-drop device. *Nat Protoc.* 2009;4(9):1328-1337.

5. Szczygielski J, Hubertus V, Kruchten E, et al. Brain Edema Formation and Functional Outcome After Surgical Decompression in Murine Closed Head Injury Are Modulated by Acetazolamide Administration. *Frontiers in neurology.* 2019;10:273.

6. Committee TUoPIACaU. IACUC guideline mouse anesthesia and analgesia recommendations. . In: Committee TUoPIACaU, ed: Spandidos Publications; 2014:3.

7. Chen Y, Constantini S, Trembovler V, Weinstock M, Shohami E. An experimental model of closed head injury in mice: pathophysiology, histopathology, and cognitive deficits. *Journal of neurotrauma.* 1996;13(10):557-568.

8. Szczygielski J, Muller A, Mautes AE, et al. Selective Brain Hypothermia Mitigates Brain Damage and Improves Neurological Outcome after PostTraumatic Decompressive Craniectomy in Mice. *Journal of neurotrauma.* 2017;34(8):1623-1635.

9. Szczygielski J, Glameanu C, Muller A, et al. Changes in Posttraumatic Brain Edema in Craniectomy-Selective Brain Hypothermia Model Are Associated With Modulation of Aquaporin-4 Level. *Frontiers in neurology.* 2018;9:799.
