# Supplementary material for: Prolonged course of brain edema and neurological recovery in a translational model of decompressive craniectomy after closed head injury in mice
Source: Front Neurol. 2023 Nov 20;14:1308683. doi: 10.3389/fneur.2023.1308683 (PMC10694459; doi:10.3389/fneur.2023.1308683)
Supplement: Supplementary file 2 [file Data_Sheet_2.pdf]

Prolonged course of brain edema and neurological recovery in a translational model of decompressive craniectomy after closed head injury in mice

SUPPLEMENTARY DATA

Supplementary material 2:

Magnetic Resonance Imaging protocol

Animals were subjected to radiological analysis according to the well-established protocol of magnetic resonance imaging (MRI), described and published in detail as an open-access protocol elsewhere (Szczygielski et al. 2018).[1] In brief, isoflurane anesthesia was induced and maintained by application of a 0.8%/99.2% to 2.5%/97.5% mixture of isoflurane and O2 via a nose cone. Vital parameters (respiration rates and cardiac rates) were continuously monitored and recorded, allowing us to adapt the intensity of anesthesia to the current condition of the animal. The temperature was maintained at 37°C by placing the animals on a special tray with an integrated heating system.

MR images were acquired using a system with a static magnetic field strength of 9.4T (Bruker BioSpec Avance III 9.4/20 with ParaVision 5.1 operating software) equipped with an actively shielded gradient. An actively detuned single channel volume coil with an inner diameter of 70 mm, a maximum peak pulse power of 1000 Ws and a maximum single pulse energy of 5 Ws served as the transmitter (in transmit-only mode). For receiving MRI signals, an actively decoupled pretuned phased array surface coil with 2x2 elements designed for imaging of the mouse brain was placed over the skull and centered over the brain midline. After placing the animal in the isocenter of the magnet, MRI was performed with a FLASH localizer sequence for identification of the symmetry axis of the brain as well as for assuring proper axial, sagittal and coronal orientation. A 3D FISP sequence in axial orientation was then used to verify correct positioning with symmetric imaging of the brain, and slice geometry data were loaded into standard Multi Slice Multi Echo (MSME, T1 weighted), Turbo Spin Echo (TSE, T2 weighted) and Echo Planar Imaging (DWI, diffusion weighted) sequences.

Both T1- and T2-weighted imaging were performed in axial orientation with a field of view of 1.76x1.50 cm2, matrix size of 234x200, slice thickness of 0.75 mm, interslice distance of 0.0 mm and number of slices of 23, generating a set of images covering the whole brain.

T1-weighted imaging was performed with TR/TE=1000/10 ms, number of excitations=4, and duration 13 min 20 s), while matching axial T2-weighted images were acquired with TR/TE=2500/30 ms, number of excitations=5, and duration 5 min 12 s.

For accurate quantification of brain tissue inflicted by edema, DWI was also performed in the axial direction with the following parameters: Field of View 1.92x1.92 cm2, Matrix Size=192x192, Slice Thickness 0.75 mm, Interslice Distance 0.0 mm, Number of Slices=7, TR/TE=2000/18.2 ms, Number of Excitations=1, Duration 48 s, B Values of 6.45 s mm-2 and 786.74, 789.19 and 789.19 s mm-2 in the sagittal, axial and coronal directions.

Brain edema was identified in T2-weighted images and ADC maps calculated from the DWI data, and matching regions of interest were manually created with the Paravision 5.1 ROI tool. The resulting size measurements (in pixels and mm2) were exported via a specially adapted macro, and the total volume of the different

lesions was calculated after importing the data into Microsoft Excel 2003® for Windows XP®.

References to Supplementary Material 2

1. Szczygielski J, Glameanu C, Muller A, et al. Changes in Posttraumatic Brain Edema in Craniectomy-Selective Brain Hypothermia Model Are Associated With odulation of Aquaporin-4 Level. *Frontiers in neurology.* 2018;9:799.
