# Supplementary material for: Prolonged course of brain edema and neurological recovery in a translational model of decompressive craniectomy after closed head injury in mice
Source: Front Neurol. 2023 Nov 20;14:1308683. doi: 10.3389/fneur.2023.1308683 (PMC10694459; doi:10.3389/fneur.2023.1308683)
Supplement: Supplementary file 3 [file Data_Sheet_3.pdf]

Prolonged course of brain edema and neurological recovery in a translational model of decompressive craniectomy after closed head injury in mice

SUPPLEMENTARY DATA

Supplementary material 3:

Histopathological processing protocol

Experimental animals were sacrificed using transcardial perfusion with buffered formaldehyde solution; the brains were removed and fixed in the same solution for 7 days. The brains were paraffin embedded, and serial coronal sections of the brains (5 µm) were made, presenting the region of interest (ROI), i.e., the coronal slices displaying hippocampal areas CA1 and CA3 as assessed using the stereotactic mouse brain atlas[1]. First, two sections were stained with hematoxylin and eosin (H&E) as well as by the Nissl staining technique. Thereafter, the slices were immunostained with anti-GFAP antibody according to the following procedure: Coronal sections were deparaffinized and rehydrated. Antigen retrieval was performed by boiling sections in 10 mM citrate buffer (pH 6.0) for 20 min. After cooling for 20 min and washing with Tris puffer, sections were incubated with 0.3% H2O2 in methanol for 20 min and washed with Tris; thereafter, intrinsic binding sites were blocked using 5% goat serum plus a biotin/avidin blocking kit (Vector Laboratories; SP-2001) and incubated with rabbit polyclonal GFAP antibody with mouse reactivity (Abcam; ab-16997, dilution 1:100) at 4 °C overnight. After washing with Tris puffer, sections were incubated with biotinylated goat anti-rabbit antibody (Vectastain Elite ABC HRP Kit (Peroxidase Rabbit IgG) Vector Laboratories; PK-6101) at room temperature for 60 min and thereafter washed with Tris puffer for 10 min. Next, sections were incubated with an avidin-biotin-peroxidase system (Vectastain Elite ABC HRP Kit (Peroxidase Rabbit IgG) Vector Laboratories; PK-6101) for 20 min and washed with Tris puffer for 10 min. Thereafter, slices were stained using a chromogen kit (Vector VIP Peroxidase HRP Substrate Kit; Vector Laboratories; SK-4600) for 20 min, washed with distilled water for 3 min and thereafter counterstained with hematoxylin.

The coronal sections underwent qualitative analysis by an independent observer blinded to the treatment of the animal utilizing a light microscope (Olympus, x40/x100/x200).

References to Supplementary Material 3

1. Paxinos G, Franklin KBJ. The Mouse Brain in Stereotaxic Coordinates. 2nd Edition ed. San Diego: Academic Press; 2001.
